# Supplementary material for: Programmable DNA protonuclei reveal environmental context on protein phase separation
Source: Nat Commun. 2026 Sep 30;17:10363. doi: 10.1038/s41467-026-78144-1 (PMC13627716; doi:10.1038/s41467-026-78144-1)
Supplement: Supplementary file 4 [file 41467_2026_78144_MOESM7_ESM.pdf]

nature portfolio

Peer Review File

Programmable DNA Protonuclei Reveal Environmental Context on Protein Phase Separation

Corresponding Author: Professor Andreas Walther

This file contains all reviewer reports in order by version, followed by all author rebuttals in order by version.

Version 0:

Reviewer comments:

Reviewer #1

(Remarks to the Author)
In this manuscript, Walther and coworkers employ their previously reported and well-characterized all-DNA core–shell coacervates as a model of the cellular nucleus, which they term a "protonucleus," to investigate protein phase separation under conditions that mimic the crowded nuclear milieu. Using a recombinant FUS construct containing GFP and MBP, the authors successfully recruit the soluble protein into the dense coacervate phase, where phase separation of FUS–GFP is triggered by TEV protease cleavage. This approach provides an elegant and effective means to localize soluble proteins within the coacervate phase and to control LLPS within a confined microenvironment. The authors further explore how changes to the coacervate composition, through the use of modified DNA sequences or crosslinked DNA, and changes to the material properties of the dense phase influence FUS–GFP recruitment and phase separation behavior. Overall, the work is elegant and conceptually appealing, offering a versatile platform to study protein aggregation and phase separation in synthetic nuclear-like environments. With the addition of several control experiments, clarifications, and improved connections to biological systems, the manuscript should be suitable for publication in Nat Commun.
1. The introduction could be more focused on the motivation for mimicking the nuclear microenvironment specifically, rather than the general cellular crowding. Including biologically relevant examples, especially those related to disease-associated nuclear protein aggregation or phase separation would strengthen the rationale.
2. FUS-GFP loading: Please clarify how the loading ratio was determined. Which method was used to quantify FUS concentration in the dense phase? Given the importance of this analysis to the work, I would expect quantification by a protein chemistry method in addition to imaging.
3. TEV cleavage: Please clarify the exact timing of enzyme addition. Figure 1 caption mentions imaging 3–4 hours after TEV addition. Does this mean that MBP-FUS-GFP was first recruited into the coacervates, followed by TEV addition, and that imaging was performed 3–4 hours post-cleavage?
4. The extent of FUS cleavage by TEV within the dense phase should be quantified. What percentage of cleavage occurs after 3–4 hours? Please indicate the fraction of cleaved protein, and verify that TEV does not alter the coacervate structure by imaging non-loaded coacervates after TEV treatment.
5. The potential effect of cleaved MBP on FUS phase behavior should be tested by a control experiment using purified MBP added to the coacervates.
6. FUS–A20 affinity: Please discuss the lack of FUS binding to A20. Given the comparable charge and aromaticity of T20 and A20, it is surprising that only T20 interacts with FUS. Could DNA conformation or specific base-group chemistry explain this difference?
7. When correlating EMSA results with protein partitioning, it seems the authors do not account for the partitioning of each modified DNA/RNA sequence. Quantifying their concentrations in both dense and dilute phases would clarify how these modifications affect FUS recruitment, e.g., a higher concentration of a modified DNA in the dilute phase could explain reduced FUS partitioning despite high affinity.
8. FRAP of FUS-GFP condensates inside the PN (presented in Figure 2g) should be compared with FRAP of FUS-GFP in bulk (not in the dense phase). This difference could highlight how the crowded dense phase of the PN affect the dynamics of FUS.
9. The unusually large error bars in the FUS–GFP $t_{1/2}$ values for non-crosslinked PNs (Figure S9) require explanation. Please include recovery plots and discuss differences from the results in Figure 2.
10. The suppression of phase separation in crosslinked PNs—particularly the partially crosslinked (25%) samples in Figure 3—deserves a deeper physicochemical explanation.
11. The caption for Figure 4 is titled "FUS–GFP in 75% crosslinked sample remains dynamic after 4 day, yet the FRAP data

are shown only for t=3 days, and recovery is minimal. ‘Dynamic’ may not be the appropriate term here.
12. The discussion could better contextualize the biological relevance of this system. Under what conditions does the viscoelasticity of the cell nucleus change in vivo? Two examples are briefly mentioned in the Conclusions section but a more expanded discussion of such examples would help clarify the implications and significance of this synthetic model.

Reviewer #2

(Remarks to the Author)
In the manuscript “DNA Protonuclei as Programmable Nuclear Mimics Reveal Environmental Context on Protein Phase Separation”, Fritzen et al investigated DNA Protonuclei (PN) as a platform for experimental model systems that mimic the nucleus for the study of protein phase separation. This work could potentially be useful to biomolecular condensate community and complement test-tube assays and complex cellular milieu. One of their findings–specifically, how FUS protein condensation is prevented in crosslinked PN compartment–provides important insight into how FUS liquid-to-solid transition occurs in cytosol. However, there are major concerns regarding the experiments and the broad conclusions they propose. These concerns and comments are listed below:

1. Authors need to clarify why PN platforms are important for protein condensation studies to target the broad audiences at Nature Communications.
a. Are there any biophysical property comparison of this PN to the nucleus, beyond the amount of DNA? Is this work based on the assumption that the amount of DNA is a critical factor for the protein condensation? In line 32-44, the authors argue that crowders are inadequate for replicating the nucleus environments because their problematic partitioning and interactions can alter physical properties of condensates. However, the ssDNA used also partitioned into the FUS condensates (Figure S3), and thus, it could potentially alter FUS condensate properties. The potential broad outcome of this study seems weakly supported.
b. Overall, controlled experiments are necessary. For example, the author should consider to test FUS proteins condensation after TEV cleavage with these DNAs in solution, or test FUS protein condensation with these DNAs in presence of crowders. These experiments are crucial to demonstrate that the PN platforms is a better experimental model for studying protein condensation under nuclear-like conditions. I understand that these experiments can be potentially challenging. In such case, a robust comparative discussion of their observations against established literature findings is critical.
2. Significant issues with FRAP measurements and data representations:
a. The FRAP method, raw images, and fitting equations are missing. This omission is critica, as standard literature practice requires showing FRAP curves alongside representative microscope images (bleached ROI and recovery progression). This representation is essential because, in extreme cases of excessive photobleaching, no meaningful recovery will be detected, irrespective of molecular diffusivity. Therefore, I would like to request the authors provide a set of FRAP images, clearly showing the bleached area and the recovery observation window. This is particularly important for the experiments where they report no recovery at all.
b. It is also important to note that the recovery kinetics can vary depending on whether partial or entire droplet bleaching is performed. If the authors intend to infer the biophysical properties of droplets inferred from the fluorescence recovery curves of the molecule of interest, I recommend they perform partial droplet bleaching and show the similar trends for different molecules. This is because entire droplet bleaching yields convoluted results, combining molecular diffusion in/out of the droplet with diffusion within the droplet itself.
c. For Figure S9, while the authors provided the half-life of the recovery and the mobile fraction, which characterize the FRAP curve shapes, it is essential to present the raw FRAP curves, the fitted curves, and the fitting equations. This full representation is necessary because the calculated half-life values are highly dependent on the fitting model applied.
d. The authors state that only three FRAP measurements were performed per data point, which is considered an inadequate number of samples and raises potential concerns. This is because FRAP results can vary significantly due to numerous factors, including droplet size, size of the region of interest (ROI), partitioning, and the identity of the tracked molecules.
3. The factors driving this partitioning trend remain unclear, especially given that RNA-protein interactions are not the major determinant.
a. The specific time point used for calculating the partition coefficient must be mentioned. This clarity is necessary because the system is shown to form distinct sub-compartments that evolve over time (as indicated in Figure S4).
b. What are these microstructures (sub-compartments), and how do they relate to the overall partitioning trned? Although PN compartment exhibits varied FUS partitioning (Figure 2d), the FUS fluorescence intensity itself (which may reflect the local concentration of FUS within these sub-compartments) appears similar across the different experimental conditions shown in Figure S4. Further explanation of these microstructures is needed to reconcile the overall partitioning data and their discrepancy with affinity experiments.
c. Could you elaborate more on how to interpretate the affinity experiment in Figure 2e? For example, why are different protein concentrations used in this affinity assay, and how are the phase separation dynamics of FUS affected by the resulting differences in local concentration and partitioning? How does it correlate to the protein diffusivity in these droplets? Figure 2f is insufficient to support the partitioning trend of FUS, but FUS protein FRAP data could be useful to substantiate the conclusion.

Minor points:
1. The authors noted that the samples were stored for several days for certain measurements. I suggest the detailed storage conditions be included in the methods section (e.g. storage vessel and sealing method). The exceptional stability of these PNs as individual droplets over such a long timeframe is valuable and presents a significant advantage for potential applications, such as using PNs as microreactors.

2. It would be important to mention how many of independent trials that they performed, and what N means in figure 2g (the number of PN compartments?).

Reviewer #3

(Remarks to the Author)
The authors proposed an LLPS study here to investigate phase separation (i.e., PS, the way they noted) of a system comprised of DNA and protein molecules. The authors used FUS-eGFP protein in tandem with a DNA-based binary phase separating model that is capable of forming core-shell condensates. By incorporating MBP domain into the FUS protein, the authors were able to control the onset of FUS condensation upon the cleavage of MBP which stands for maltose binding protein, using TEV protease. This would allow the authors to have FUS-eGFP dispersed inside the core-shell condensates to ensure that FUS condensation occurs inside the DNA condensates. Along with a brief investigation into the aging of FUS condensates, the authors also characterized the thermodynamics of the system mainly by functionalizing the DNA subunits to interact with FUS and drive its condensation.
The manuscript is well-written, the language is solid, and the message is conveyed. It benefits from a decent, potentially interesting idea that focuses on localizing proteins with the ability to phase separate into synthetic DNA condensates. The authors did a fair job depicting the results and discussing them. Nevertheless, the manuscript does not offer a deep, fully characterized study on this DNA-protein intertwined model, and it falls short on providing enough quantitative results. In specific, the authors did not methodically prove some of their claims, including the phase behavior of the FUS condensates, to the extent that some of the findings are contradictory (see the comments below). They also named their intertwined condensates model proto-nucleus which is biologically incorrect and irrelevant. In line with my comments below, I did not find the paper suitable for publication in Nature Communications, as it needs foundational changes and revisions.

- In the opening sentences of the Introduction, the authors brought examples of natural condensates, such as nucleolus, Cajal bodies, paraspeckles, and nuclear speckles in the cell nucleus. Later they called their PS model protonucleus which sounds odd to the common knowledge one has for the nucleus and does not suitably represent that. I found this labeling quite unfit, and the problem is that the authors have built the core of their study based on this flawed definition. I would strongly advise that they clarify and change this assimilation of their binary condensate model to nucleus. Moreover, it is barely convincing that the so-called protonucleus platform is an advanced and versatile test system between classical experiments and in cellulo works (according to the authors words in lines 48 and 49). I cannot see the proper extent to which the authors expanded the viability of this proposed platform beyond FUS, so their definition again seems unfit.
- At first glance, the DNA concentration span used in this study is hardly comparable to and overlapping with that of the nucleus. I don't understand the authors' reasoning to make such a claim while it is obvious that the two ranges are not quite comparable. This lack of comparability is also the case when it comes to the size of the condensates. According to Figure 2d, I can see the majority of size distributions for virtually all the experiments done with the modification sequences are well below the line specified for human cell nucleus. This claim has to either be mainly revised or eliminated from the manuscript.
- How did the authors determine the Droplet Domain in Figure 1? From the image shown, I cannot deduce whether the 0.37 condition is actually in droplet domain, which implies the liquid behavior of the FUS condensates. To me, this condition actually cannot be perceived as droplet phase, rather it is more akin to a percolated gel morphology. This might arise from the microscopy artifact. Regardless, unless FRAP measurements are performed, it is not correct to use the term droplet for the condensates.
- Comparing the results in Figures 1 and 2, I wonder if the observed morphologies due to the addition of modified strands can be reproduced by changing the loading ratio only. The authors did not provide any long-term images of different loading ratios, nor did they fully investigate the entire span (gaps between 0.17 and 0.31, 0.37 and 0.61, and 0.72 and 1.16). It seems that two modifications, cases 5 and 6, yield nearly identical morphology, that is concentric green shell, to that of the pristine condition. So, this begs the question that this extra modification might not even be necessary. The authors must have drawn the limitations of the pristine condition and its loading ratio tuning before trying to use modifications that seemingly complicate the system. I understand that the morphologies in cases 3 and 4 are quite different and distinct. Nevertheless, the authors did not confirm that those morphologies cannot be achieved simply by tuning the loading ratio.
- The authors picked the loading ratio of 0.3 for a good portion of the experiments. Why did this ratio was not used in their phase diagram? Also, this seemingly random selection of ratios begs the question that why the authors left such big gaps between the ranges of each domain they defined in Figure 1? They defined the Droplet Domain (although unconvincingly), yet they did not adhere to that range. The reasoning provided (line 115) is that the extra nucleic acid compared to FUS is ensured to be achieved. However, this itself is not convincing, especially when I compare 0.31 ratio shown in Figure 1 and 0.3 ratio shown in Figure 2c for the pristine condition which should be the same as those in Figure 1. The condensation regimes in 0.3 (5 h) and 0.31 (3-4 h) are nothing alike and hardly even similar. I can see quite big condensates with a shell for 0.3 (Figure 2c), while only small condensates are visible for 0.31 (Figure 1, second from left). What is the justification here to explain this odd difference, and if this significant difference appears in the course of 1-2 h, the authors should discuss and demonstrate it further.
- In case 4, T20^{atto647}, how is it that this complementary domain unlike the shell molecules, p(T20-n), does not coat the DNA condensates and form a shell-like interface between the dilute and dense phase? I am not sure why this added strand can diffuse in and hybridize to the ploy-A in the core instead of accumulating across the outer surface of the condensates. In fact, the 2-days images reveal that this condition is the most uniformly distributed one inside the core. Moreover, comparing that with case 2, m*^{atto647} is surprising given the almost opposite effect they have for the FUS partitioning. Is this due to a specific interaction of FUS with the m domains of the p(A20-n) molecules? The authors need to investigate this further and also measure the partitioning of added strands in addition to FUS, e.g., partition coefficient of T20^{atto647} vs. m*^{atto647} could give us an insight into the factors that contributed to the different partitioning of FUS, which is currently not discernable based on the EMSA results.
- I think the time frame for both FRAP measurements shown in Figure 2 seems insufficient. I find it hard to conclude if the

recovery is slow or nonexistent. Providing time constant for the recovery curves and elongating the recovery monitoring time can help. Also, why did the authors not perform FRAP experiments after the FUS addition and before TEV protease? I can see in Figure 2g that the FUS behavior is more dynamic, but I am also wondering if FUS presence in the dispersed phase inside the condensates might change the recovery curves of the core channel. In any case, I cannot safely say that FUS condensates are much more dynamic than the core condensates given that the recovery in the span of 60 s is only about 20% and it very much looks like it reached plateau.
- The same can be said to some extent about the FRAP data shown in the last figure, where the authors discussed aging of these condensates. I believe a longer FRAP observation along with calculating the time constant (tau) would be essential to help interpret the results and consolidate the claims. I cannot tell whether the sample with 75% crosslinkers remains dynamic after 4 days. The FRAP data shows FUS dispersed, condensed after 6 h, and condensed after 3 days, but not 4 days (Figure 4). The authors should confirm whether it is 3 days (FRAP curve) or 4 days (figure caption) that is the point of discussion. Regardless, the recovery curve after 3 days does not suggest a notable dynamic (showing only 10% recovery with a semi-plateau curve after 50 s).
- I am not sure what to expect from Figure 3, as it is merely a visual comparison of the samples with varied concentration of the self-complementary cross-linker. The figure contains big fields of view with large pool of condensates, though it is quite difficult to compare the cases even qualitatively given that the image sizes are not consistent. The kinetics of these samples need to be investigated further and a comparative analysis of the dynamics should be provided, so that the authors can support their claim about the great reduction in the dynamics of the condensates by adding p(A20-m-XL) both in terms of morphology and time progression (lines 227 and 227).

- What is the repeating units of p(A20-m) based on which the authors defined the loading ratio? It needs to be discussed more clearly.
- For the Soluble Domain vs. Exceeding Loading Capacity, I recommend the authors add partition coefficient calculations to clearly show the difference. Even though the green domain inside the shell is brighter for the second case, it needs to be supported by partition coefficient or a similar measurement.
- What are the details about the EMSA experiments, such as concentrations used, the incubation time of the FUS and the target nucleic acid strands, voltage etc.?
- The authors need to quantify the colocalization that seems to exist in Figures S2 and S3. The current presentation of the data is purely based on qualitative comparison, and while it can be seen, the colocalization needs to be shown by appropriate processing tools.
- On top of page 11 of the SI, the authors noted “Figure S4 shows” while I believe it should be Figure S6

Reviewer #4

(Remarks to the Author)
I co-reviewed this manuscript with one of the reviewers who provided the listed reports. This is part of the Nature Communications initiative to facilitate training in peer review and to provide appropriate recognition for Early Career Researchers who co-review manuscripts.

Version 1:

Reviewer comments:

Reviewer #1

(Remarks to the Author)
I find the revised manuscript to be a significantly improved version of the work. The authors have addressed my previous concerns and performed the necessary analyses to eliminate remaining ambiguities. In particular, the partition coefficient analysis and the MD simulations of T20 and A20 binding to FUS are informative. The TEV cleavage analysis addresses my earlier comment, and the control FRAP experiments are an important addition to the study. Overall, in my view, this is a well-rounded work that is suitable for publication in Nature Communications.

Reviewer #2

(Remarks to the Author)
I appreciate the reviewer's detailed clarifications and the additional work they put into addressing my comments. All of my concerns have been fully resolved, and I have no further questions.

Reviewer #3

(Remarks to the Author)
I appreciate the effort that went into the revision, it is clear that the authors made a serious effort to respond: the manuscript is stronger and more coherent. In particular, the authors rebuilt the loading-ratio analysis, added a continuum-model framework in new Figures 2–3, and clarified some of the prior technical ambiguities regarding loading-ratio definition and EMSA conditions. I have a few remaining recommendations:
1) Changing the wording from a nucleus-mimetic system to “nucleus-inspired” is a step in the right direction. However, the title still foregrounds “protonuclei,” and the Introduction still presents PN as “an advanced and versatile test system between classical test tube/solution experiments and in cellulo studies” (lines 63-70). This still overstates the biological analogy. I

recommend either softening the nomenclature further or explicitly narrowing these platform-level claims throughout the manuscript.
2) The new Figure 1 has some discrepancies from the previous version. The morphologies of the condensates are drastically different between the two versions. For instance, for the ratios 0.30 (new), the condensates appear larger in size after 5 hours compared to the ratio 0.37 (old) after 3-4 hours. The same morphological difference is true for the other regimes. More importantly, the “Exceeding Loading Capacity” is no longer observed in the new version and instead, “Inverted Region” is reported as the third regime for ratio 1.50. This level of inconsistency is concerning and the authors did not discuss it neither in the response nor in the manuscript. Moreover, in the revised manuscript, the whole notion of exceeding loading capacity is omitted. Was that observation wrong or was not reproducible? The authors need to clarify this. The same is true for the “Co-continuous Domain” that is now replaced by “Spinodal Domain” entirely without much similarity between the morphologies. In the rebuttal, the authors mentioned that they removed “Exceeding Loading Capacity” (page 22), though the authors need to clarify why they did not use HEPES for Figure 1 in the first place.
3) I still think that Figure 5 (formerly Figure 3) should include some quantification about the variability of the morphologies and condensate sizes with respect to the p(A20-m) ratios. I understand that the authors provided FRAP data to support the claims quantitatively, though the measure of size and morphology variations remain unanswered.
4) The revised Figure 6 addresses the earlier 3-day versus 4-day inconsistency and is much more informative than before. Nevertheless, terms such as “remains dynamic after 3 days” (the caption of the figure) are somewhat stronger than I would prefer given that the recovery remains “partial recovery” (as the authors added) rather than fully liquid-like behavior. Also, the new Figure 4 does not include the requested post-loading/pre-cleavage FRAP control, so the paper still cannot fully exclude the possibility that FUS loading changes core mobility before TEV addition. I recommend reducing the wording to “retains partial mobility” where appropriate and refine what the FRAP controls establish.

Reviewer #4

(Remarks to the Author)
I co-reviewed this manuscript with one of the reviewers who provided the listed reports. This is part of the Nature Communications initiative to facilitate training in peer review and to provide appropriate recognition for Early Career Researchers who co-review manuscripts.

Open Access This Peer Review File is licensed under a Creative Commons Attribution 4.0 International License, which permits use, sharing, adaptation, distribution and reproduction in any medium or format, as long as you give appropriate credit to the original author(s) and the source, provide a link to the Creative Commons license, and indicate if changes were made.
In cases where reviewers are anonymous, credit should be given to 'Anonymous Referee' and the source.
The images or other third party material in this Peer Review File are included in the article’s Creative Commons license, unless indicated otherwise in a credit line to the material. If material is not included in the article’s Creative Commons license and your intended use is not permitted by statutory regulation or exceeds the permitted use, you will need to obtain permission directly from the copyright holder.
To view a copy of this license, visit https://creativecommons.org/licenses/by/4.0/

Reviewer comments:

Reviewer 1:

In this manuscript, Walther and coworkers employ their previously reported and well-characterized all-DNA core–shell coacervates as a model of the cellular nucleus, which they term a “protonucleus,” to investigate protein phase separation under conditions that mimic the crowded nuclear milieu. Using a recombinant FUS construct containing GFP and MBP, the authors successfully recruit the soluble protein into the dense coacervate phase, where phase separation of FUS–GFP is triggered by TEV protease cleavage. This approach provides an elegant and effective means to localize soluble proteins within the coacervate phase and to control LLPS within a confined microenvironment. The authors further explore how changes to the coacervate composition, through the use of modified DNA sequences or crosslinked DNA, and changes to the material properties of the dense phase influence FUS–GFP recruitment and phase separation behavior. Overall, the work is elegant and conceptually appealing, offering a versatile platform to study protein aggregation and phase separation in synthetic nuclear-like environments. With the addition of several control experiments, clarifications, and improved connections to biological systems, the manuscript should be suitable for publication in Nat Commun.

Thank you for your positive evaluation of our manuscript!

1. The introduction could be more focused on the motivation for mimicking the nuclear microenvironment specifically, rather than the general cellular crowding. Including biologically relevant examples, especially those related to disease-associated nuclear protein aggregation or phase separation would strengthen the rationale.

We thank the reviewer for this insightful comment and have revised the Introduction to more clearly articulate why we specifically mimic the nuclear microenvironment, and to ground this choice in biologically and disease-relevant examples.

First, we now emphasize that the nucleus is not merely a crowded compartment, but a distinct phase-separating environment characterized by high DNA/RNA content, transcriptional activity, and viscoelastic chromatin constraints. In the revised Introduction (paragraph 1), we explicitly highlight nuclear condensates such as nucleoli, Cajal bodies, paraspeckles, and nuclear speckles as protein–nucleic-acid assemblies whose formation and material properties are governed by these nuclear-specific features. This distinguishes our motivation from generic macromolecular crowding in the cytoplasm.

Added text:

The nucleus presents a unique environment comprising numerous RNA/protein- and chromatin-based condensates whose assembly and material properties are governed by high nucleic-acid content, transcriptional activity, and viscoelastic chromatin constraints.[4,5]

Second, we added disease-relevant nuclear examples to motivate our focus on nuclear, rather than general cellular, phase separation.

Added text:

Importantly, several RNA-binding proteins implicated in neurodegenerative diseases, such as amyotrophic lateral sclerosis (ALS) and frontotemporal dementia (FTD), including Fused in sarcoma (FUS) and TDP-43, form nuclear condensates *in vivo*[15] and occasionally form pathological aggregates in the nucleus.[16] FUS undergoes PS at sites of DNA double-strand breaks, where FUS-dependent PS is required for the recruitment of DNA repair factors and higher-order clustering of chromatin nanodomains. This identifies chromatin-associated, nucleic acid-rich and mechanically constrained nuclear environments as critical regulators of FUS condensate function.[17]

We deliberately kept the biological motivation focused on FUS and closely related nuclear condensates, as this provides direct disease-relevance of our study. At the same time, the broader utility of the PN system lies in its programmability: nucleic-acid composition, compartmentalized architecture, confinement, and viscoelasticity can be tuned independently in a bottom-up manner. We believe this combination of a focused biological model and a modular experimental framework provides a clear and coherent rationale for the present work.

2. FUS-GFP loading: Please clarify how the loading ratio was determined. Which method was used to quantify FUS concentration in the dense phase? Given the importance of this analysis to the work, I would expect quantification by a protein chemistry method in addition to imaging.

In the original version of the manuscript, the loading ratio (**LR**) was defined operationally as the molar ratio of FUS added to the sample relative to the repeating units of p(A_{20}-m), and protein concentrations inside protonuclei were not independently quantified. Because several

reviewer comments highlighted ambiguities in the interpretation of Figure 1, we decided to substantially revise and strengthen this analysis. We have implemented the following changes:

1. We have now used the same buffer for Figure 1 as used in all other experiments (HEPES buffer: 10 mM HEPES, 200 mM KCl, 1 mM DTT, 5 mM $MgCl_2$, Glycerol 3%, pH 7.5).
2. We now exactly match LR 0.30 of Figures 4-6.
3. We have recorded the initial PS process (first 30 min) as video (Supplementary Movie 1), and have documented the PS process over 5 h.
4. We have imaged larger frames containing multiple PN and added the overview images to the SI (Supplementary Fig. 6).

Since our revised experiments include larger populations of PN, we were able to determine the partitioning of MBP-FUS-GFP before TEV addition. This analysis shows a sharp increase in partitioning from 0.10 to 0.30 LR, followed by the maximum saturation at 0.30 LR. The mean partition coefficient of **P** = 448 closely matches the value in Figure 4d (old figure 2d), proving robust batch-to-batch reliability of our system. With higher LR the partitioning decreases, which originates from a saturation effect: While the absolute amount of FUS inside PN is higher at LR 1.5, the relative amount is slightly less than for LR 0.30.

This is represented well by plotting LR x P against the LR, giving an “enrichment-weighted loading”. In case [FUS] reaches a maximum loading in the PN, the plot of LR x P should turn constant, which is not yet the case in our experimental window. This now explains the continuous change in PS morphologies with LR. These further analyses are shown below and have been added as Supplementary Fig. 4.

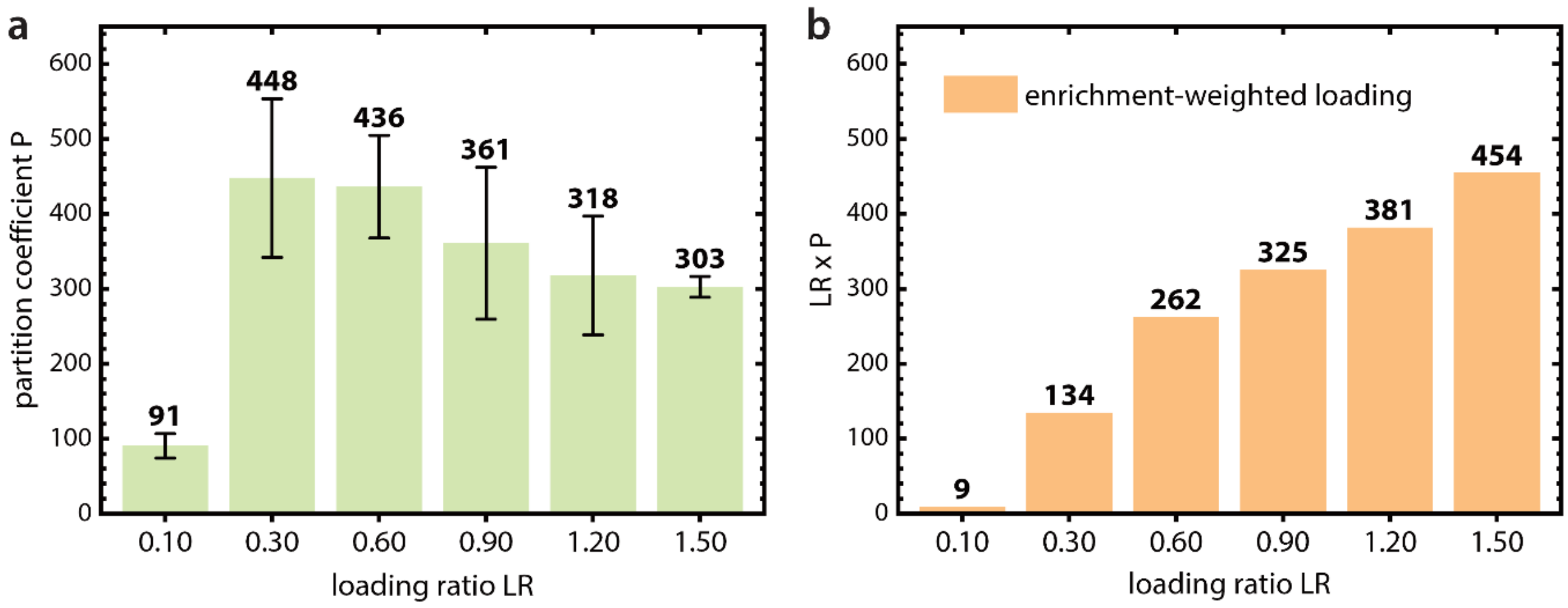

3. TEV cleavage: Please clarify the exact timing of enzyme addition. Figure 1 caption mentions imaging 3–4 hours after TEV addition. Does this mean that MBP-FUS-GFP was first recruited into the coacervates, followed by TEV addition, and that imaging was performed 3–4 hours post-cleavage?

Yes, the experiment was conducted as described by the reviewer, and as outlined in Scheme 1c. However, in line with other reviewer comments we have decided to repeat the experiments in Figure 1 to follow the PS process over time. You now find a revised version of Figure 1 and additional data in the SI file (Supplementary Fig. 4-6). We also provide Supplementary Movie 1 showing the PS process over the first 30 minutes.

4. The extent of FUS cleavage by TEV within the dense phase should be quantified. What percentage of cleavage occurs after 3–4 hours? Please indicate the fraction of cleaved protein, and verify that TEV does not alter the coacervate structure by imaging non-loaded coacervates after TEV treatment.

We provide a TEV cleavage assay in the SI file (see Supplementary Fig. 17), showing instantaneous and complete cleavage of the MBP tag after less than two minutes. This is still well below the first time points in Figure 4c (old Figure 2c), which show the state at 5 min after cleavage.

Additionally, we measured the influence of TEV, free MBP and the combination of both on pristine PN samples. There is no impact on the morphology of PN and no PS in any scenario. (see Supplementary Fig. 1-3)

5. The potential effect of cleaved MBP on FUS phase behavior should be tested by a control experiment using purified MBP added to the coacervates.

As explained above, we have run your suggested control experiment and have not seen PS in these samples (see Supplementary Fig. 1-3).

6. FUS–A20 affinity: Please discuss the lack of FUS binding to A20. Given the comparable charge and aromaticity of T20 and A20, it is surprising that only T20 interacts with FUS. Could DNA conformation or specific base-group chemistry explain this difference?

Thank you for raising this interesting point. T and A are in fact different regarding their aromaticity (purine vs. pyrimidine base). To address it we collaborated with Prof. Lukas Stelzl for all-atom MD simulations of T20 and A20 binding to the RGG2 domain of FUS. The results are included in SI Section 11 (Supplementary Fig. 15 and 16) and show that A20 has such strong intramolecular base-stacking that its available conformations for binding to the protein are limited. For T20 this is less the case, which is why binding is stronger for it in silico and in our experiments.

7. When correlating EMSA results with protein partitioning, it seems the authors do not account for the partitioning of each modified DNA/RNA sequence. Quantifying their concentrations in both dense and dilute phases would clarify how these modifications affect FUS recruitment, e.g., a higher concentration of a modified DNA in the dilute phase could explain reduced FUS partitioning despite high affinity.

To quantify the amount of DNA and RNA binding to the core material, we have calculated the melting curves for all core-modifications with NUPACK (version 4.0.2.0).

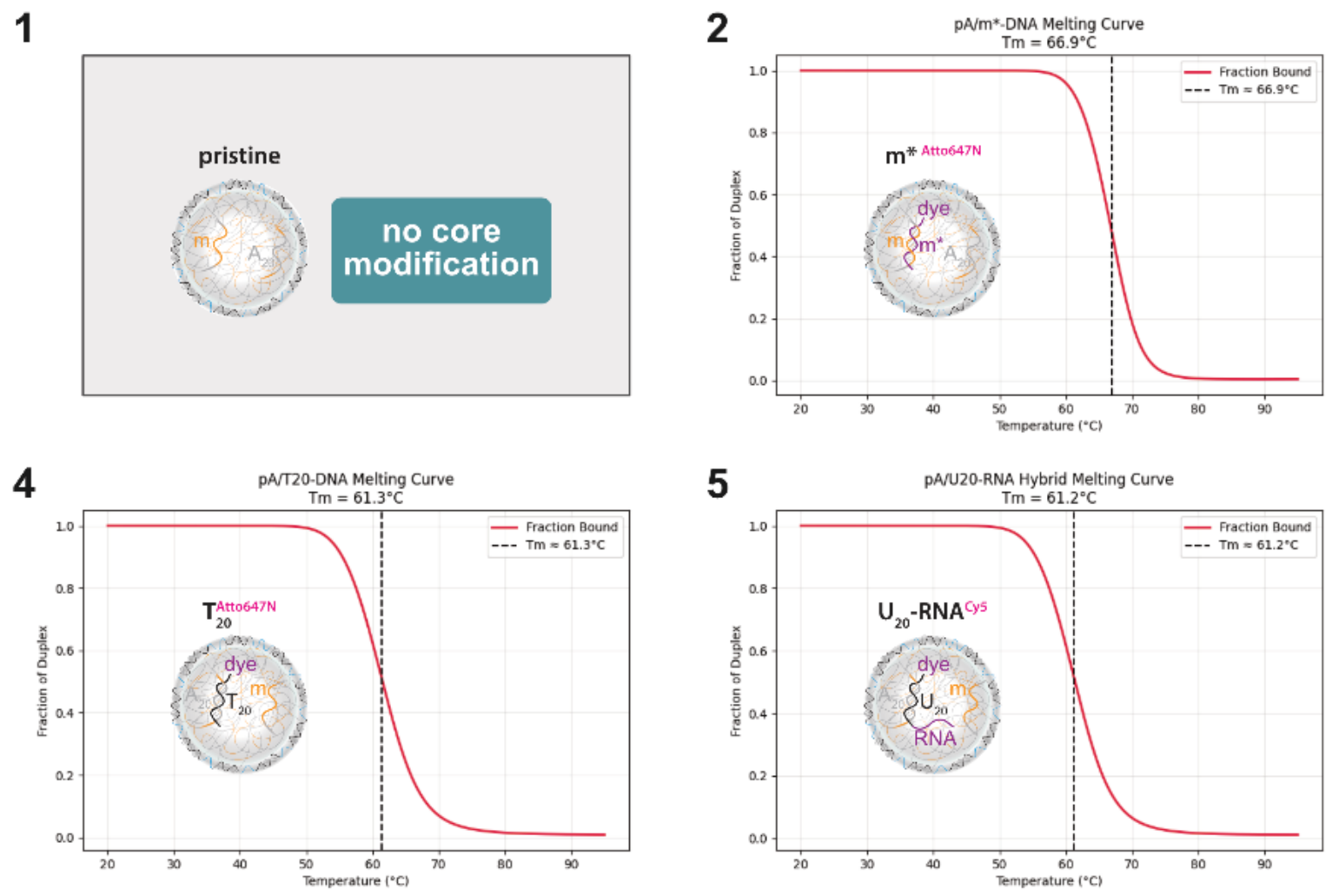

The results show that all core modifications are tightly anchored in the PN by their dsDNA binding sequences under the experimental conditions (25°C, buffer (salt), and concentration). Since core modification strands are used in sub-stoichiometric amounts (80%), we can be sure they are fully bound within the PN. This analysis and discussion has now been included as Supplementary Fig. 7.

8. FRAP of FUS-GFP condensates inside the PN (presented in Figure 2g) should be compared with FRAP of FUS-GFP in bulk (not in the dense phase). This difference could highlight how the crowded dense phase of the PN affect the dynamics of FUS.

We assume the reviewer wants to know differences in FRAP behavior between FUS droplets in buffer and of FUS/nucleic acid droplets in PN. We now include FRAP data of FUS obtained in newly performed droplet assays in Figure 6a.

Please note, the point of Figure 4g (former 2g) is to underline the liquid properties of FUS after 1 day, which can facilitate the merging seen in the snapshots in Figure 4c (former 2c).

9. The unusually large error bars in the FUS–GFP $t_1/_2$ values for non-crosslinked PNs (Figure S9) require explanation. Please include recovery plots and discuss differences from the results in Figure 2.

We believe the reviewer was referring to the large error bars in the mobile fraction from original Figure S9 (now Supplementary Fig. 21). To improve transparency, we have included the combined average trajectories of the raw traces and the resulting fits into the figure with the fit data. In addition, we have adjusted the reporting of FRAP characteristics according to reviewer feedback. Since not all recovery plots reach saturation within the observation window, we now refer in these cases to “recovery intensity after x seconds” or endpoint recovery (ER) instead of mobile fraction. Both alternatives use normalized intensity value after the defined time, which allows us to compare samples without inferring knowledge of the mobile fraction based on partial recovery fits.

With this new reporting the error bars for the recovered intensity after 30 seconds remain relatively large for pristine PN, reflecting the bigger variation in the trajectories being averaged in Supplementary Fig. 21a. The data shows a trend of decreased error with slower recovery. This can be explained by higher fitting sensitivity of quickly recovering samples, as less data points cover the initial slope. Hence these samples are more sensitive to laser instabilities, detector noise or sample drift. An additional factor can be diffusion during the bleaching process, which is relevant since the CLSM is scanning the bleaching area sequentially.
Lastly, the comparison with the FRAP results in Figure 2 (now Figure 4) does not seem appropriate to us, since Supplementary Fig. 21 compares the diffusivity of FUS within crosslinked PN, whereas the FRAP experiments in Figure 4f investigate the dynamics of the PN core material. However, on a morphological basis (Figure 4c vs Figure 5) it is clear, that a dynamic asymmetry between PN core and FUS can lead to viscoelastic phase separation (VPS), as discussed to great detail in the MS.

10. The suppression of phase separation in crosslinked PNs—particularly the partially crosslinked (25%) samples in Figure 3—deserves a deeper physicochemical explanation.

The key physicochemical point is that crosslinking converts the PN core into a more slowly relaxing, stress-bearing network. Formation and growth of FUS-rich domains then require local rearrangement and deformation of this DNA matrix, which becomes increasingly hindered as crosslink density rises. As a result, phase separation is progressively slowed and mechanically frustrated: at 25% p(A_{20}-m-XL), FUS demixing can still occur locally, but coarsening and reorganization are strongly arrested, leading to the observed spongy/network-like morphology. At higher crosslinking, visible phase separation is suppressed on the experimental timescale and resolution.

Added text:

The XL motifs impose additional connectivity within the PN core, thereby increasing the energetic and kinetic cost of redistributing the DNA core during FUS condensation. This restricts domain growth and relaxation, so that the 25% crosslinked sample remains trapped in a finely structured, spongy morphology rather than progressing toward larger, coarsened FUS-rich phases.

11. The caption for Figure 4 is titled "FUS–GFP in 75% crosslinked sample remains dynamic after 4 day, yet the FRAP data are shown only for t=3 days, and recovery is minimal. 'Dynamic' may not be the appropriate term here.

We have clarified this mismatch by repeating the experiments (new Figure 6) and aligning both experiments to t=3 days. Also, we increased the time of FRAP recovery observation (from 45 s in old Figure 4, to 120 s in new Figure 6), now yielding more substantial recovery that justifies the term "dynamic" in our eyes.

12. The discussion could better contextualize the biological relevance of this system. Under what conditions does the viscoelasticity of the cell nucleus change in vivo? Two examples are

briefly mentioned in the Conclusions section but a more expanded discussion of such examples would help clarify the implications and significance of this synthetic model.

We thank the reviewer for this suggestion. We agree that nuclear viscoelasticity can change in vivo, for example through variations in chromatin organization or nuclear mechanics, and we already briefly mention such cases in the Conclusions.

However, the aim of our study is not to directly reproduce specific cellular states, but to isolate how key physical parameters such as viscoelasticity, confinement, and multivalency influence phase separation in a controlled system. The protonucleus therefore serves as a minimal model that complements, rather than mimics, the full cellular complexity.

We have clarified this point in the Discussion and emphasize that our main conclusion is the general role of the physical environment as a regulator of condensate behavior. We have added & restructured the following text in the Conclusions:

The ability of PN to suppress PS or alter condensate morphology via crosslinking parallels how nuclear viscoelasticity regulates biological function *in vivo*. For instance, mechanical deformation during confined cell migration induces chromatin reorganization that can trigger condensate coalescence and de novo assembly.[67] Furthermore, nuclear stiffness is dynamically tuned by chromatin compaction, which suppresses PS in stiff heterochromatin regions and allows PS in softer euchromatin regions. By using targeted condensation and stiffness gradients, it is assumed that cell nuclei can dynamically restructure their genomic loci. [68] This modulation of nuclear PS in cells points to potential physical regulators of phase transitions beyond biochemical interactions.

Reviewer 2:

In the manuscript "DNA Protonuclei as Programmable Nuclear Mimics Reveal Environmental Context on Protein Phase Separation", Fritzen et al investigated DNA Protonuclei (PN) as a platform for experimental model systems that mimic the nucleus for the study of protein phase separation. This work could potentially be useful to biomolecular condensate community and complement test-tube assays and complex cellular milieu. One of their findings–specifically, how FUS protein condensation is prevented in crosslinked PN compartment–provides important insight into how FUS liquid-to-solid transition occurs in cytosol. However, there are major concerns regarding the experiments and the broad conclusions they propose. These concerns and comments are listed below:

We thank the reviewer for the careful evaluation of our work and for recognizing the potential of the PN platform to complement existing approaches. We appreciate the critical comments raised and have addressed them in detail below. We have revised the manuscript accordingly, added clarifications, and expanded the analysis where necessary. We hope that these changes resolve the concerns and improve the clarity and robustness of the study, and that the revised version will be considered suitable for publication.

1. Authors need to clarify why PN platforms are important for protein condensation studies to target the broad audiences at Nature Communications.

a. Are there any biophysical property comparison of this PN to the nucleus, beyond the amount of DNA? Is this work based on the assumption that the amount of DNA is a critical factor for the protein condensation? In line 32-44, the authors argue that crowders are inadequate for replicating the nucleus environments because their problematic partitioning and interactions can alter physical properties of condensates. However, the ssDNA used also partitioned into the FUS condensates (Figure S3), and thus, it could potentially alter FUS condensate properties. The potential broad outcome of this study seems weakly supported.

Our study is not based on the assumption that DNA concentration alone governs protein condensation. Instead, the central aim of the PN platform is to capture how nucleic acid density, multivalency, confinement, and material state jointly regulate protein phase behavior, factors that are largely inaccessible in dilute solution or crowder-based assays.

Beyond matching nuclear DNA concentrations, PN reproduce several key biophysical features relevant to nuclear condensation: (i) high nucleic acid density within micron-scale confinement, (ii) multivalent and sequence-defined interaction networks, and (iii) tunable viscoelasticity via programmable DNA crosslinking. Notably, altering the mechanical state of the PN core progressively suppresses FUS phase separation and delays liquid-to-solid transitions, highlighting the importance of material properties in regulating condensate behavior. We have lightly expanded the introduction to account for some aspects.

We agree with the reviewer that ssDNA partitions into FUS condensates (Supplementary Fig. 10). Importantly, this does not undermine our argument but rather reflects a hallmark of

nuclear environments, where nucleic acids are active, co-condensing components rather than inert background. The key distinction from polymeric crowders is that DNA in PN is chemically defined, spatially confined, and experimentally controllable, allowing us to systematically dissect how co-partitioning affects condensate morphology, dynamics, and aging.

Crucially, our results show that DNA/RNA sequences with comparable solution affinities produce dramatically different partitioning and phase behaviors inside PN, demonstrating that protein condensation cannot be predicted from affinity or concentration alone. This highlights the central message of our work: environmental context, not just molecular binding, fundamentally reshapes protein phase separation.

b. Overall, controlled experiments are necessary. For example, the author should consider to test FUS proteins condensation after TEV cleavage with these DNAs in solution, or test FUS protein condensation with these DNAs in presence of crowders. These experiments are crucial to demonstrate that the PN platforms is a better experimental model for studying protein condensation under nuclear-like conditions. I understand that these experiments can be potentially challenging. In such case, a robust comparative discussion of their observations against established literature findings is critical.

We thank the reviewer for this suggestion and have now performed the proposed control experiments. We examined FUS phase separation after TEV cleavage in solution-based droplet assays with the same DNA and RNA binders used in the PN experiments (Supplementary Fig. 22). In all cases, FUS formed indistinguishable liquid droplets, with no discernible differences in morphology or behavior between strong and weak binders, providing no additional mechanistic insight beyond droplet formation itself.

We further tested FUS condensation in the presence of PEG as a molecular crowder. Strikingly, MBP–FUS already phase separated prior to TEV cleavage, demonstrating the well-known non-innocent behavior of PEG and its ability to directly perturb protein phase behavior.

Together, these results underscore that bulk droplet assays and crowders obscure sequence- and context-dependent effects that are readily resolved in PN. In contrast, PN allow controlled protein recruitment prior to phase separation and reveal environment-dependent behaviors that remain inaccessible in solution-based assays.

2. Significant issues with FRAP measurements and data representations:

a. The FRAP method, raw images, and fitting equations are missing. This omission is critica, as standard literature practice requires showing FRAP curves alongside representative microscope images (bleached ROI and recovery progression). This representation is essential because, in extreme cases of excessive photobleaching, no meaningful recovery will be detected, irrespective of molecular diffusivity. Therefore, I would like to request the authors provide a set of FRAP images, clearly showing the bleached area and the recovery observation window. This is particularly important for the experiments where they report no recovery at all.

We have included a general paragraph about FRAP evaluation in the SI, including fitting equations and exemplary raw images for reference (see Supplementary Fig. 13, Supplementary Equations (12)-(14)). We now provide the FRAP curves for all measurements in this manuscript. As the reviewer points out, we also included the recovery observation images for the samples showing no recovery to the SI (Supplementary Fig. 14).

b. It is also important to note that the recovery kinetics can vary depending on whether partial or entire droplet bleaching is performed. If the authors intend to infer the biophysical properties of droplets inferred from the fluorescence recovery curves of the molecule of interest, I recommend they perform partial droplet bleaching and show the similar trends for different molecules. This is because entire droplet bleaching yields convoluted results, combining molecular diffusion in/out of the droplet with diffusion within the droplet itself.

All FRAP data shown here uses partial FRAP, as seen by the now provided recovery observation images (see for instance Supplementary Fig. 13).

c. For Figure S9, while the authors provided the half-life of the recovery and the mobile fraction, which characterize the FRAP curve shapes, it is essential to present the raw FRAP curves, the fitted curves, and the fitting equations. This full representation is necessary because the calculated half-life values are highly dependent on the fitting model applied.

We have now added the full representation of the FRAP investigation in this revision (see Supplementary Fig. 13, Supplementary Equations (12)-(14)).

d. The authors state that only three FRAP measurements were performed per data point, which is considered an inadequate number of samples and raises potential concerns. This is because FRAP results can vary significantly due to numerous factors, including droplet size, size of the region of interest (ROI), partitioning, and the identity of the tracked molecules.

We thank the reviewer for raising this point. We agree that a larger number of FRAP measurements would in principle be desirable. However, in our system, FRAP experiments are time-intensive and must be performed on the same sample over extended periods. Importantly, in several samples FUS condensates evolve over time – which is a tracking parameter in the system (e.g., coarsening and aging) – such that prolonged data acquisition or large numbers of sequential measurements would compromise comparability. For this reason, we limited the analysis to a small number of measurements per condition, performed on the same sample that is also shown in the corresponding figures, ensuring consistency between imaging and FRAP analysis.

3. The factors driving this partitioning trend remain unclear, especially given that RNA-protein interactions are not the major determinant.

a. The specific time point used for calculating the partition coefficient must be mentioned. This clarity is necessary because the system is shown to form distinct sub-compartments that evolve over time (as indicated in Figure S4).

We thank the reviewer for highlighting the need for clarity. The partition coefficients were quantified after equilibration of MBP–FUS–GFP within the protonuclei but prior to TEV-induced cleavage and the onset of FUS phase separation, when the PN interior is homogeneous and no sub-compartments are present. Thus, the reported values reflect protein recruitment into the PN environment, not redistribution caused by phase separation or subsequent structural evolution. We mention in the MS text, Figure 4d in-figure text and figure legend that the partitioning is measured for MBP-FUS-GFP. Additionally, we now clarify the time point in the MS text with the following addition in yellow highlight:

We first quantify the partitioning of MBP-FUS-GFP into PN before initiation of PS by comparing the mean GFP intensity inside the PN core with that of the solution (background; Figure 4d).

b. What are these microstructures (sub-compartments), and how do they relate to the overall partitioning trned? Although PN compartment exhibits varied FUS partitioning (Figure 2d), the FUS fluorescence intensity itself (which may reflect the local concentration of FUS within these sub-compartments) appears similar across the different experimental conditions shown in Figure S4. Further explanation of these microstructures is needed to reconcile the overall partitioning data and their discrepancy with affinity experiments.

The observed microstructures (sub-compartments) arise after TEV-induced cleavage and reflect sequence- and material-dependent phase separation pathways of FUS within the confined PN environment, including droplet formation, co-continuous networks, and shell-associated condensates. Importantly, these structures do not inform the partition coefficients, which were quantified under homogeneous conditions, prior to phase separation.

The apparent similarity in local FUS fluorescence intensity within individual sub-compartments therefore does not contradict the partitioning data. The partition coefficient reports the global enrichment of FUS in the PN relative to the surrounding solution, whereas the fluorescence intensity within condensates reflects local concentration after phase separation, which can converge across conditions due to saturation within the dense phase.

This decoupling explains why affinity measurements and partitioning trends diverge: solution affinity governs binary binding, while PN partitioning and subsequent microstructure formation are controlled by multivalency, confinement, and material properties of the DNA-rich environment.

c. Could you elaborate more on how to interpretate the affinity experiment in Figure 2e? For example, why are different protein concentrations used in this affinity assay, and how are the phase separation dynamics of FUS affected by the resulting differences in local concentration and partitioning? How does it correlate to the protein diffusivity in these droplets? Figure 2f

is insufficient to support the partitioning trend of FUS, but FUS protein FRAP data could be useful to substantiate the conclusion.

In Figure 4e (former 2e), we used two FUS concentration regimes: a higher concentration to compare binding across all DNA duplexes under identical conditions, and a lower concentration to demonstrate that the RNA binder interacts with FUS at substantially lower protein concentrations, consistent with its known high affinity. Importantly, the EMSA is intended as a qualitative/semi-quantitative ranking of binary binding in dilute solution and is not used to infer phase separation dynamics.

Regarding the link to partitioning and phase separation: in PN, both local concentration and phase behavior emerge from recruitment into a confined, multivalent environment, so the relationship between solution affinity, enrichment, and condensate morphology is not one-to-one. This is exactly why we observe cases where sequences with similar EMSA shifts lead to strongly different PN partitioning and phase separation pathways.

We agree that FUS FRAP can provide additional support. However, a systematic comparison across all PN conditions is experimentally challenging because FUS adopts distinct, evolving morphologies (droplets, arrested networks, shell-associated condensates), which makes defining comparable ROIs and time points nontrivial and risks conflating diffusion with aging and restructuring. We therefore used FRAP in Figure 4f (former 2f) solely to quantify core material dynamics as an environmental control parameter, and included an example FUS FRAP measurement (Figure 4g, former 2g) to show that FUS can remain mobile even when the PN core is effectively immobile.

Further FRAP data and comparison to pure FUS droplets can be found in the revised Figure 6 (old Figure 4) and the corresponding SI (Supplementary Fig. 22).

Minor points:

1. The authors noted that the samples were stored for several days for certain measurements. I suggest the detailed storage conditions be included in the methods section (e.g. storage vessel and sealing method). The exceptional stability of these PNs as individual droplets over such a long timeframe is valuable and presents a significant advantage for potential applications, such as using PNs as microreactors.

We need to distinguish between PN and the FUS-PN samples.

FUS-PN: All samples that were monitored over multiple days were stored directly in the imaging well plate at room temperature (25 °C). After addition of TEV protease, the plate was sealed to prevent evaporation, and no further buffer exchange was performed during the experiment.

PN: PN stock solutions were generally used immediately after preparation. The only exception was the second batch of Figure 1 experiments (loading ratios 0.9–1.5), where PN stocks in high-salt buffer were stored at 4 °C for three days prior to use. Under these conditions, PN integrity and behavior were indistinguishable from freshly prepared samples.

More generally, we routinely observe that PN stock solutions in high-salt buffer remain stable for weeks to months, highlighting their exceptional robustness. We have now included these storage details in the Methods section and note that this stability represents a practical advantage of PN for long-term studies.

2. It would be important to mention how many of independent trials that they performed, and what N means in figure 2g (the number of PN compartments?).

We believe the reviewer meant to say Figure 2**d** (now 4**d**). In this figure, N denotes the number of individual protonuclei (PN compartments) within a given CLSM frame that were used for the statistical analysis of diameter and partition coefficients. We clarified this in the caption and also added a paragraph for replication and statistical reporting to the SI.

Reviewer 3:

The authors proposed an LLPS study here to investigate phase separation (i.e., PS, the way they noted) of a system comprised of DNA and protein molecules. The authors used FUS-eGFP protein in tandem with a DNA-based binary phase separating model that is capable of forming core-shell condensates. By incorporating MBP domain into the FUS protein, the authors were able to control the onset of FUS condensation upon the cleavage of MBP which stands for maltose binding protein, using TEV protease. This would allow the authors to have FUS-eGFP dispersed inside the core-shell condensates to ensure that FUS condensation occurs inside the DNA condensates. Along with a brief investigation into the aging of FUS condensates, the authors also characterized the thermodynamics of the system mainly by functionalizing the DNA subunits to interact with FUS and drive its condensation.

We thank the reviewer for this accurate summary of our experimental strategy.

The manuscript is well-written, the language is solid, and the message is conveyed. It benefits from a decent, potentially interesting idea that focuses on localizing proteins with the ability to phase separate into synthetic DNA condensates. The authors did a fair job depicting the results and discussing them. Nevertheless, the manuscript does not offer a deep, fully characterized study on this DNA-protein intertwined model, and it falls short on providing enough quantitative results. In specific, the authors did not methodically prove some of their claims, including the phase behavior of the FUS condensates, to the extent that some of the findings are contradictory (see the comments below). They also named their intertwined condensates model proto-nucleus which is biologically incorrect and irrelevant. In line with my comments below, I did not find the paper suitable for publication in Nature Communications, as it needs foundational changes and revisions.

We thank the reviewer for the careful evaluation and for acknowledging the clarity of the manuscript and the conceptual interest of the approach. In response to the reviewer's comments, we have added new control experiments, expanded quantitative analyses, and revised the text to more clearly define the scope, limitations, and strengths of the PN platform. We believe these revisions directly address the concerns raised and substantially strengthen the manuscript without altering its conceptual foundation.

- In the opening sentences of the Introduction, the authors brought examples of natural condensates, such as nucleolus, Cajal bodies, paraspeckles, and nuclear speckles in the cell nucleus. Later they called their PS model protonucleus which sounds odd to the common knowledge one has for the nucleus and does not suitably represent that. I found this labeling quite unfit, and the problem is that the authors have built the core of their study based on this flawed definition. I would strongly advise that they clarify and change this assimilation of their binary condensate model to nucleus. Moreover, it is barely convincing that the so-called protonucleus platform is an advanced and versatile test system between classical experiments and in cellulo works (according to the authors words in lines 48 and 49). I cannot see the

proper extent to which the authors expanded the viability of this proposed platform beyond FUS, so their definition again seems unfit.

We would like to clarify that we do not claim biological equivalence between protonuclei and the cell nucleus, nor do we intend the term protonucleus as a literal representation of nuclear structure or function. Instead, the term is used deliberately in analogy, similar to widely accepted concepts such as protocells or artificial organelles, to denote minimal, programmable, bottom-up model systems.

The motivation for introducing protonuclei is precisely to avoid overinterpretation of native nuclear systems while gaining experimental control over key physical parameters: nucleic acid density, confinement, multivalency, and viscoelasticity. These are central to nuclear phase separation but difficult to disentangle in cells or nuclear extracts. In this sense, PN are positioned between dilute in vitro assays and in cellulo studies, not as substitutes for either, but as a controllable intermediate that allows systematic interrogation of environmental effects. We note that the two other reviewers did not oppose the notion of terming these species protonuclei.

To address the reviewer's concerns, we have changed the terminology from "nucleus-mimetic" to "nucleus-inspired".

Regarding generality beyond FUS, we emphasize that FUS is used here as a well-established model nucleic-acid–binding, disease-relevant, phase-separating protein to demonstrate the capabilities of the platform. The PN system itself is protein-agnostic: its programmability lies in the DNA-based environment rather than in protein-specific design.

- At first glance, the DNA concentration span used in this study is hardly comparable to and overlapping with that of the nucleus. I don't understand the authors' reasoning to make such a claim while it is obvious that the two ranges are not quite comparable. This lack of comparability is also the case when it comes to the size of the condensates. According to Figure 2d, I can see the majority of size distributions for virtually all the experiments done with the modification sequences are well below the line specified for human cell nucleus. This claim has to either be mainly revised or eliminated from the manuscript.

Please see our comment just above.

Regarding DNA concentration, PN span 5–13 g/L, whereas reported nuclear DNA concentrations range from approximately 9–31 g/L, depending on cell type and nuclear volume assumptions. While this does not represent full overlap, the values are clearly within the same order of magnitude, which was the intended basis of comparison. We have reworded the sentence to take into account the slightly smaller size of PN vs cell nuclei: "The DNA concentration is 5-13 g/L, which approximates that of the nucleus in a cell of about 9-31 g/L (Supplementary Table 2)."

Regarding size, PN exhibit mean diameters of ~3.7–4.7 µm, whereas a typical human cell nucleus has an average diameter of ~6 µm. We agree that PN are generally smaller than nuclei, and we now describe them as "slightly smaller but comparable in scale", rather than "similar" without qualification. Importantly, both size and concentration are meant to establish physical scale, not structural identity.

Crucially, the conclusions of our study do not rely on exact matching of nuclear dimensions or DNA concentrations, but on the ability of PN to provide a confined, DNA-rich, and mechanically tunable environment in which context-dependent phase behavior can be isolated and studied systematically.

- How did the authors determine the Droplet Domain in Figure 1? From the image shown, I cannot deduce whether the 0.37 condition is actually in droplet domain, which implies the liquid behavior of the FUS condensates. To me, this condition actually cannot be perceived as droplet phase, rather it is more akin to a percolated gel morphology. This might arise from the microscopy artifact. Regardless, unless FRAP measurements are performed, it is not correct to use the term droplet for the condensates.

We thank the reviewer for this important clarification. The assignment of the "droplet domain" in original Figure 1 was based on morphological criteria from CLSM images, and we agree that the exact transition between droplet-like and other morphologies cannot be defined with absolute precision. For this reason, we deliberately depicted the boundaries between domains in Figure 1 as blurred and phenomenological, rather than as sharp phase boundaries, to avoid implying a strict classification.

Importantly, the onset of the spinodal regime is more clearly identifiable. It is characterized by the appearance of a well-defined exclusion zone adjacent to the PN shell, corresponding to a characteristic length scale of composition modulation; a classic hallmark of spinodal decomposition reflecting a system-specific wavelength. We also observe this behavior in simulations of the system, supporting its interpretation as spinodal-like phase separation.

Finally, in the revised experiments we also observe an inverted regime, in which droplets of the dilute phase appear within a continuous FUS-rich phase, further supporting that Figure 1 captures a continuous slice through the phase diagram.

To further underscore the phase separation processes, we now provide numerical simulations based on expanded Flory Huggins theory to map phase diagrams and binodal and spinodal regime. This data has been added to the new Figures 2 and 3, including discussion of the model used for the simulations. This modeling approach now substantially underpins the conclusions drawn from Figure 1.

Revised figure, based on new experiments:

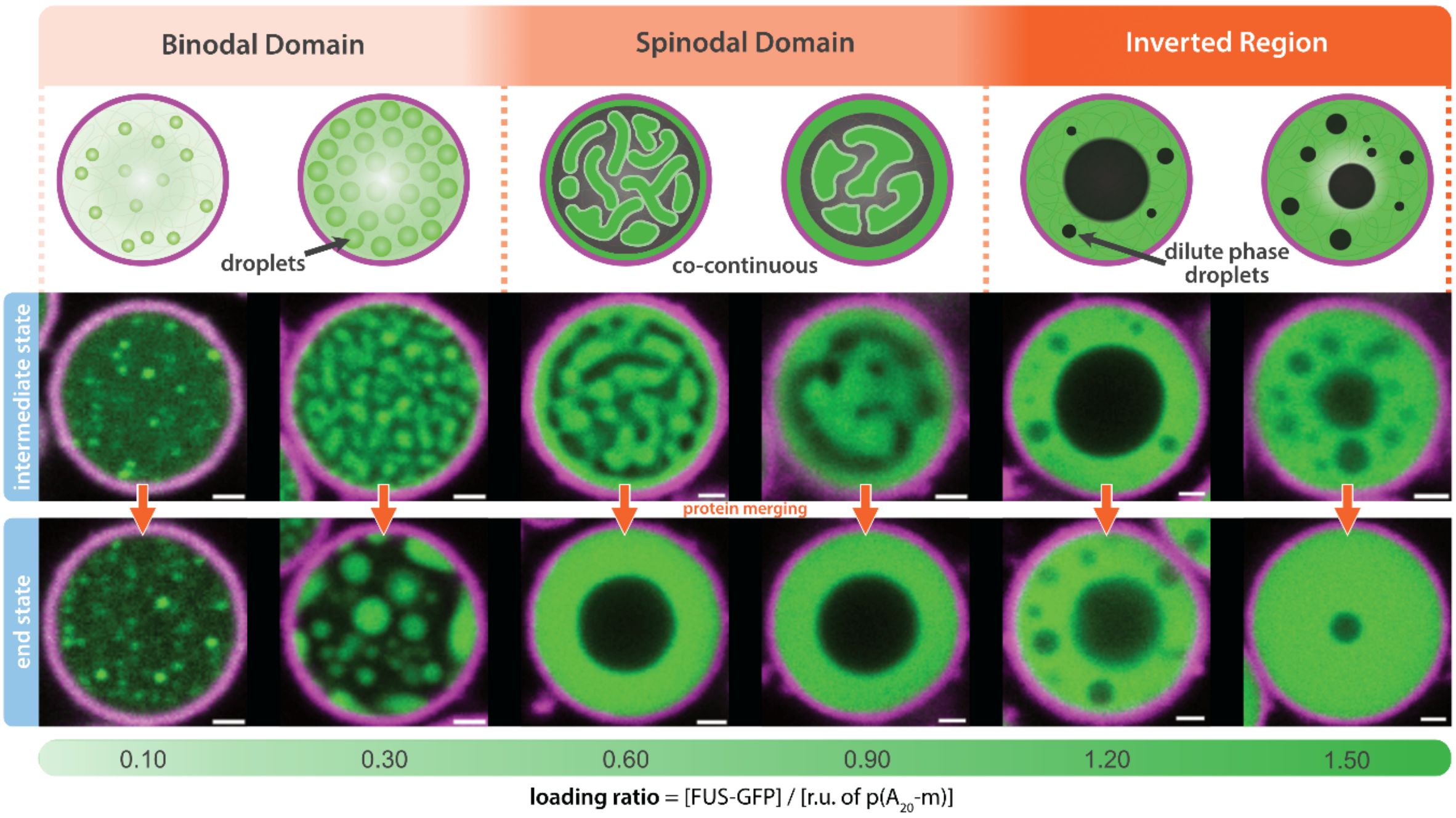

- Comparing the results in Figures 1 and 2, I wonder if the observed morphologies due to the addition of modified strands can be reproduced by changing the loading ratio only. The authors did not provide any long-term images of different loading ratios, nor did they fully investigate the entire span (gaps between 0.17 and 0.31, 0.37 and 0.61, and 0.72 and 1.16). It seems that two modifications, cases 5 and 6, yield nearly identical morphology, that is concentric green shell, to that of the pristine condition. So, this begs the question that this extra modification might not even be necessary. The authors must have drawn the limitations of the pristine condition and its loading ratio tuning before trying to use modifications that seemingly complicate the system. I understand that the morphologies in cases 3 and 4 are quite different and distinct. Nevertheless, the authors did not confirm that those morphologies cannot be achieved simply by tuning the loading ratio.

We agree that protein loading ratio is an important control parameter and that, in principle, tuning the loading ratio can influence both local concentration and phase separation morphology. Indeed, Figure 1 demonstrates that varying the loading ratio alone already leads to distinct morphological regimes.

However, the key point of our study is that loading ratio and nucleic acid composition are not equivalent control parameters. While loading ratio primarily adjusts the amount of protein present, core modifications additionally alter the interaction landscape and the dynamic/mechanical properties of the DNA-rich environment. These changes directly affect recruitment, phase separation pathways, and long-term evolution in ways that cannot be captured by protein concentration alone.

The apparent similarity between some final morphologies (e.g., cases 5 and 6 versus pristine PN) does not imply equivalence of the underlying processes. In these cases, the routes to the final state, the kinetics, intermediate structures, and material dynamics differ substantially, as evidenced by altered partitioning, retention, and viscoelastic behavior. Thus, the modifications are not introduced to create unique endpoint morphologies per se, but to decouple environmental parameters from protein loading and reveal how they reshape phase behavior.

We agree that a complete, high-resolution mapping of all intermediate loading ratios and long-term morphologies would be valuable, but this is beyond the scope of the present study.

- The authors picked the loading ratio of 0.3 for a good portion of the experiments. Why did this ratio was not used in their phase diagram? Also, this seemingly random selection of ratios begs the question that why the authors left such big gaps between the ranges of each domain they defined in Figure 1? They defined the Droplet Domain (although unconvincingly), yet they did not adhere to that range. The reasoning provided (line 115) is that the extra nucleic acid compared to FUS is ensured to be achieved. However, this itself is not convincing, especially when I compare 0.31 ratio shown in Figure 1 and 0.3 ratio shown in Figure 2c for the pristine condition which should be the same as those in Figure 1. The condensation regimes in 0.3 (5 h) and 0.31 (3-4 h) are nothing alike and hardly even similar. I can see quite big condensates with a shell for 0.3 (Figure 2c), while only small condensates are visible for 0.31 (Figure 1, second from left). What is the justification here to explain this odd difference, and if this significant difference appears in the course of 1-2 h, the authors should discuss and demonstrate it further.

We thank the reviewer for raising this point and have addressed it directly by extending the loading-ratio analysis. We have now repeated the Figure 1 experiments across a continuous range of loading ratios from 0.10 to 1.50, using the same HEPES buffer conditions as in the remainder of the manuscript.

The loading ratio of 0.30 was selected for subsequent experiments because it lies in a robust intermediate regime where FUS is efficiently recruited, phase separation can be reproducibly triggered, and the system is sensitive to changes in nucleic acid composition and material properties. Importantly, under the now unified HEPES buffer conditions (for new Figure 1), the morphologies observed at 0.30 and 0.31 are consistent when compared at matched time points, resolving the earlier apparent discrepancy noted by the reviewer.

- In case 4, T20^{atto647}, how is it that this complementary domain unlike the shell molecules, p(T20-n), does not coat the DNA condensates and form a shell-like interface between the dilute and dense phase? I am not sure why this added strand can diffuse in and hybridize to the ploy-A in the core instead of accumulating across the outer surface of the condensates. In fact, the 2-days images reveal that this condition is the most uniformly distributed one inside the core. Moreover, comparing that with case 2, m*^{atto647} is

surprising given the almost opposite effect they have for the FUS partitioning. Is this due to a specific interaction of FUS with the m domains of the p(A20-n) molecules? The authors need to investigate this further and also measure the partitioning of added strands in addition to FUS, e.g., partition coefficient of T20^{atto647} vs. m*^{atto647} could give us an insight into the factors that contributed to the different partitioning of FUS, which is currently not discernable based on the EMSA results.

The key distinction is that p(T_{20}-n) is a long, multivalent polymer that kinetically arrests at the core–solution interface during PN formation, whereas $T_{20}^{Atto647N}$ is a short, monovalent oligonucleotide. As a result, $T_{20}^{Atto647N}$ readily diffuses into the PN core and hybridizes throughout the p(A_{20}-m) matrix, rather than becoming trapped at the interface, explaining its uniform distribution at long times.

To quantitatively assess binding of all core-modification strands, we performed NUPACK (v4.0.2.0) simulations of the melting curves for all DNA and RNA core modifications under the experimental salt and nucleic acid concentrations. These simulations show that all core-modification strands remain fully bonded as duplex at experimental conditions (25 °C, salinity, concentrations). Because the modification strands are added in sub-stoichiometric amounts (80%) relative to available binding sites, it follows that they are fully bound to the core material, ruling out partial hybridization or strand exchange as the origin of the observed differences.

To directly address the reviewer's question of whether a specific FUS–m interaction underlies the difference between samples **2** and **4**: this was indeed one of our initial hypotheses, and it was one motivation for designing sample **6**, which effectively increases the concentration of accessible m domains via the dangling m overhang on T_{20}-$m^{Atto647N}$. If stronger FUS–m interactions alone were the dominant determinant of recruitment, sample **6** should be expected to partition FUS at least as strongly as, or more strongly than, sample **4**. However, this is not what we observe. Despite the additional dangling m strand, sample 6 shows **lower** partitioning than $T_{20}^{Atto647N}$-PN (**4**), and PS sets in earlier. Together with the EMSA data (now Figure 4e), this argues against a simple explanation based solely on specific FUS–m binding. Rather, it supports the central conclusion of our study: isolated 1:1 affinity measurements in solution are not sufficient to predict partitioning behavior in PN with multivalent settings and conformational restrictions. Instead, recruitment emerges from the broader multivalent and confined context of the system, in which sequence identity, spatial presentation, and the dynamic state of the nucleic acid environment jointly shape FUS behavior.

- I think the time frame for both FRAP measurements shown in Figure 2 seems insufficient. I find it hard to conclude if the recovery is slow or nonexistent. Providing time constant for the recovery curves and elongating the recovery monitoring time can help. Also, why did the authors not perform FRAP experiments after the FUS addition and before TEV protease? I can see in Figure 2g that the FUS behavior is more dynamic, but I am also wondering if FUS presence in the dispersed phase inside the condensates might change the recovery curves of

the core channel. In any case, I cannot safely say that FUS condensates are much more dynamic than the core condensates given that the recovery in the span of 60 s is only about 20% and it very much looks like it reached plateau.

The purpose of Figure 2f (old)/4f is to demonstrate a strong difference in dynamics of the PN core material across core-modification conditions (mobile in the m*Atto647N case vs. effectively immobile in the others), rather than to resolve very slow diffusion with high precision. The relative difference is important and to our opinion clearly discernible in the presented data.

For Figure 2g (old)/4g, we agree that the ~20% recovery within the observation window reflects restricted mobility rather than "fully liquid-like" behavior. We have therefore adjusted the wording to avoid overinterpretation and now describe this as "partial" recovery consistent with residual exchange/mobility of FUS within the co-segregated phase, while the core channel remains essentially non-recovering under the same conditions. The intended conclusion is thus comparative: FUS retains measurable mobility on these timescales, whereas the PN core does not, consistent with an arrested DNA-rich environment that still permits limited protein exchange.

We believe that these statements are well reflected in the data. We agree that maximizing FRAP timescales is always beneficial, and we have focused our efforts here on Figure 6.

- The same can be said to some extent about the FRAP data shown in the last figure, where the authors discussed aging of these condensates. I believe a longer FRAP observation along with calculating the time constant (tau) would be essential to help interpret the results and consolidate the claims. I cannot tell whether the sample with 75% crosslinkers remains dynamic after 4 days. The FRAP data shows FUS dispersed, condensed after 6 h, and condensed after 3 days, but not 4 days (Figure 4). The authors should confirm whether it is 3 days (FRAP curve) or 4 days (figure caption) that is the point of discussion. Regardless, the recovery curve after 3 days does not suggest a notable dynamic (showing only 10% recovery with a semi-plateau curve after 50 s).

We thank the reviewer for this important comment regarding the FRAP analysis and the interpretation of condensate aging. To address these concerns, we have repeated the aging and FRAP experiments with a new batch of samples, focusing on the 0% and 75% crosslinked PN conditions.

In the revised experiments, we have unified the experimental design such that all aging-related measurements, including the RNA-invader experiments, were performed after exactly 3 days for both crosslinking conditions. This removes the previous ambiguity between 3-day and 4-day time points, and the figure caption and text have been corrected accordingly (in new Figure 6).

To better capture slow recovery processes and distinguish between very limited mobility and saturation effects, we have extended the FRAP acquisition window to 120–180 seconds, allowing us to follow the recovery curves to (nearly) plateau.

- I am not sure what to expect from Figure 3, as it is merely a visual comparison of the samples with varied concentration of the self-complementary cross-linker. The figure contains big fields of view with large pool of condensates, though it is quite difficult to compare the cases even qualitatively given that the image sizes are not consistent. The kinetics of these samples need to be investigated further and a comparative analysis of the dynamics should be provided, so that the authors can support their claim about the great reduction in the dynamics of the condensates by adding p(A20-m-XL) both in terms of morphology and time progression (lines 227 and 227).

We thank the reviewer for this comment and would like to clarify the intended purpose of Figure 3 (now Figure 5). This figure is not meant to provide a quantitative kinetic comparison, but rather to illustrate the qualitative, threshold-like effect of increasing PN crosslinking on FUS phase separation within PN. The large fields of view were deliberately chosen to demonstrate the global suppression of coarsened phase separation events as the fraction of p(A_{20}-m-XL) increases.

At low and intermediate crosslinking, FUS undergoes visible phase separation with arrested or slowly evolving morphologies, whereas at ≥ 75% crosslinking no microscopically detectable phase separation occurs over several days, despite identical protein loading and experimental conditions. This qualitative absence of coarsened structures is itself a robust and reproducible outcome that does not require further kinetic fitting to support the central conclusion.

Quantitative information on the underlying dynamics of the environment is provided independently by FRAP measurements of MBP-FUS-GFP (Supplementary Fig. 21), which show a pronounced decrease in mobility with increasing crosslinking. New Figure 5 (old Figure 3) should therefore be interpreted together with these FRAP data: the former reports on whether and how FUS phase separation manifests macroscopically, while the latter quantifies the viscoelastic constraints imposed by the crosslinked DNA matrix.

To better clarify the intention of this analysis, we have included the following sentence:

Complementary to this quantitative mobility analysis, Figure 5 shows a morphology progression over time. Together, these datasets offer a comparative analysis of the FUS PS dynamics upon PN core crosslinking.

- What is the repeating units of p(A20-m) based on which the authors defined the loading ratio? It needs to be discussed more clearly.

We thank the reviewer for pointing out that this definition could be made more explicit. The repeating unit of p(A20-m) refers to one A20 block connected to a ssDNA m barcode sequence, as generated by rolling circle amplification of the corresponding template. The loading ratio is therefore defined as the molar ratio of FUS to these p(A20-m) repeating units. We have added a reference from Figure 1 caption to Scheme 1a, where the repeating unit is specified.

- For the Soluble Domain vs. Exceeding Loading Capacity, I recommend the authors add partition coefficient calculations to clearly show the difference. Even though the green domain inside the shell is brighter for the second case, it needs to be supported by partition coefficient or a similar measurement.

We thank the reviewer for this suggestion. In the revised experiments, using uniform HEPES buffer conditions and an expanded, continuous range of loading ratios, we no longer observe a clear separation into a "soluble" versus an "exceeding loading capacity" domain based on morphology alone. Accordingly, we have removed this domain classification from the manuscript to avoid overinterpretation.

Importantly, we now provide direct quantitative partition coefficient measurements as a function of loading ratio (shown in Supplementary Fig. 4). These data demonstrate that FUS partitioning into PN increases strongly at low-to-intermediate loading ratios and then progressively decreases at higher loading, consistent with approaching saturation of the PN environment. Thus, the difference between low and high loading conditions is now supported by partition coefficient analysis rather than visual brightness alone, addressing the reviewer's concern.

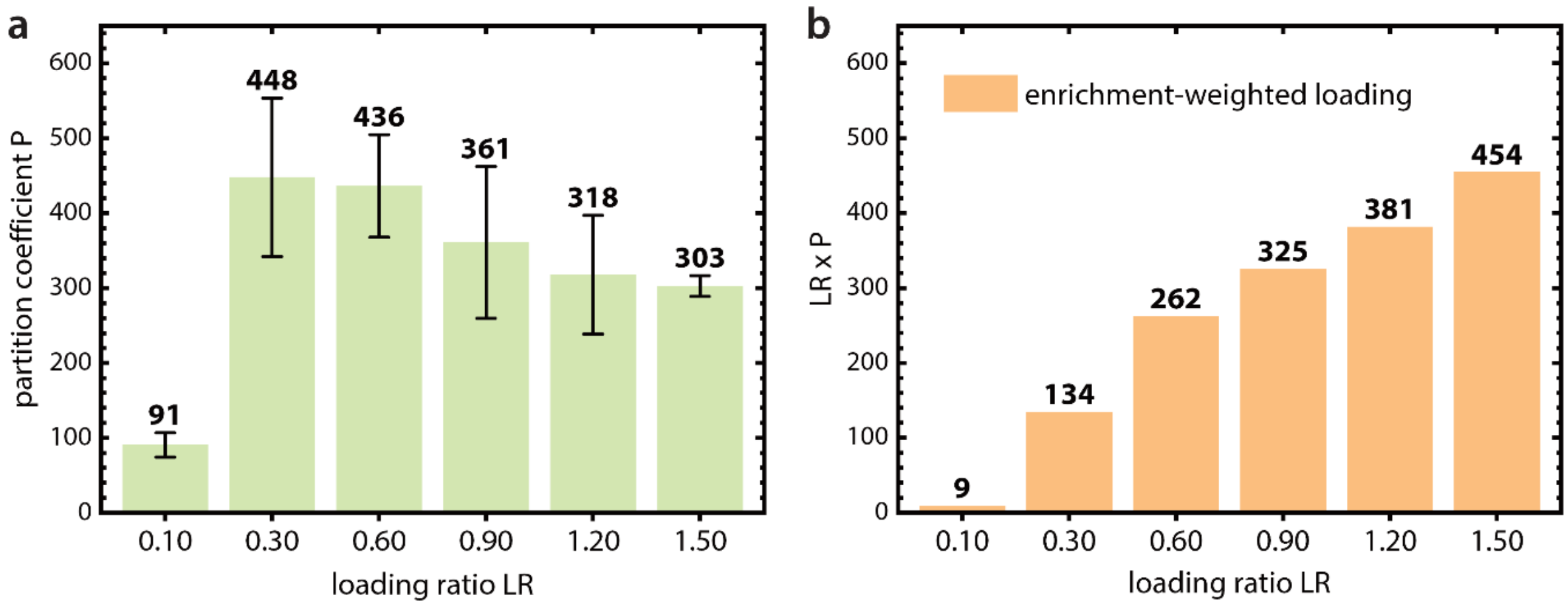

- What are the details about the EMSA experiments, such as concentrations used, the incubation time of the FUS and the target nucleic acid strands, voltage etc.?

The experimental details of the EMSA, including nucleic acid and protein concentrations, incubation time, buffer composition, gel percentage, voltage, and run time, had been provided in the Methods section (Electrophoretic Mobility Shift Assay).

- The authors need to quantify the colocalization that seems to exist in Figures S2 and S3. The current presentation of the data is purely based on qualitative comparison, and while it can be seen, the colocalization needs to be shown by appropriate processing tools.

As suggested by the reviewer we have quantified colocalization between former Figures S2 and S3 (now Supplementary Fig. 9 and 10). We have calculated the Pearson (a) and Manders (b) coefficients based on the raw image data and with the Fiji plugin JACoP. We did not use pre-determined ROIs for the analysis. The following figure compares both coefficients for all core-modified samples (not for pristine PN) before TEV cleavage and after 2 days. We also show the corresponding cytofluorograms after 2 days (c).

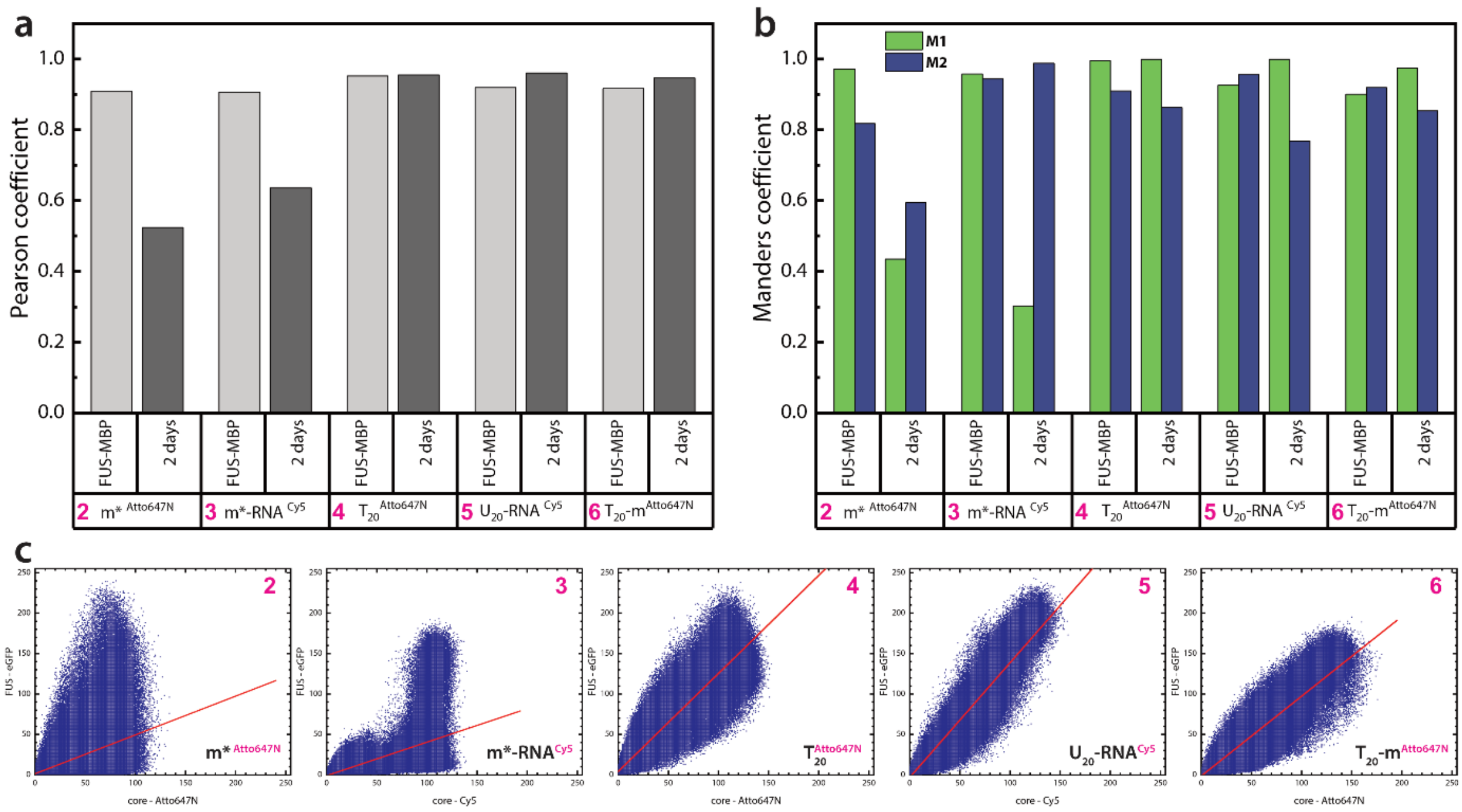

This quantification confirms the qualitative interpretation already presented in the manuscript. In the FUS-MBP state, all samples show high Pearson (a) and Manders (b) values, consistent with the expected homogeneous partitioning of soluble MBP-FUS into the PN core before TEV cleavage. After 2 days, samples **4**-**6** retain high colocalization, in agreement with their observed end-state morphologies, where either little visible phase separation occurs (**4**) or the PN core material is strongly co-segregated with the FUS condensates (**5**-**6**). By contrast, **2** and **3** show reduced colocalization at the endpoint, consistent with the fact that the core does not fully collapse into one common condensate with FUS. For **2**, this is further in line with the visible leakage of FUS into the surrounding solution. For sample **3**, the asymmetric Manders coefficients indicate that FUS is largely confined to core-positive regions, whereas only a smaller fraction of the core signal overlaps with FUS, consistent with localized FUS condensates within the core rather than complete co-collapse.

Overall, this additional analysis supports the interpretation already derived from the microscopy images, but does not provide substantial new mechanistic insight beyond the existing analyses. For this reason, we have not included these data in the revised manuscript

or Supplementary Information, in order to keep the data presentation focused. Since Nature Communication operates with a transparent peer review, this data would be available in this answer letter for the interested reader.

- On top of page 11 of the SI, the authors noted “Figure S4 shows” while I believe it should be Figure S6

Thank you. We corrected the mistake.

Reviewer 4:

I co-reviewed this manuscript with one of the reviewers who provided the listed reports. This is part of the Nature Communications initiative to facilitate training in peer review and to provide appropriate recognition for Early Career Researchers who co-review manuscripts.

We thank the reviewer for their time and effort in evaluating our manuscript.

Reviewer comments:

Reviewer 1:

I find the revised manuscript to be a significantly improved version of the work. The authors have addressed my previous concerns and performed the necessary analyses to eliminate remaining ambiguities. In particular, the partition coefficient analysis and the MD simulations of T20 and A20 binding to FUS are informative. The TEV cleavage analysis addresses my earlier comment, and the control FRAP experiments are an important addition to the study. Overall, in my view, this is a well-rounded work that is suitable for publication in Nature Communications.

We thank the reviewer for the positive evaluation of the revised manuscript and for recognizing the additional analyses and control experiments. We are pleased that the reviewer considers the manuscript suitable for publication in Nature Communications.

Reviewer 2:

I appreciate the reviewer's detailed clarifications and the additional work they put into addressing my comments. All of my concerns have been fully resolved, and I have no further questions.

We thank the reviewer for the careful assessment and are pleased that the revisions have fully resolved the previous concerns.

Reviewer 3:

I appreciate the effort that went into the revision, it is clear that the authors made a serious effort to respond: the manuscript is stronger and more coherent. In particular, the authors rebuilt the loading-ratio analysis, added a continuum-model framework in new Figures 2–3, and clarified some of the prior technical ambiguities regarding loading-ratio definition and EMSA conditions.

We thank the reviewer for the constructive re-evaluation of our manuscript and for acknowledging the substantial revisions. We address the remaining recommendations point by point below.

I have a few remaining recommendations:

1) Changing the wording from a nucleus-mimetic system to “nucleus-inspired” is a step in the right direction. However, the title still foregrounds “protonuclei,” and the Introduction still presents PN as “an advanced and versatile test system between classical test tube/solution experiments and in cellulo studies” (lines 63-70). This still overstates the biological analogy. I recommend either softening the nomenclature further or explicitly narrowing these platform-level claims throughout the manuscript.

This is a matter of semantics, chosen by us in analogy to wordings of protocell, artificial cell or synthetic cell. None of the present-day systems in these fields have the complexity and functions of any real living cell when looked at these systems through the eyes of a biologist. However, they all (mostly) do mimic selected essential features. The same rational holds for choosing the word “protonucleus”. It shall imply some level of analogy, without being certainly able to replicate all the intricate details in structure and composition of a real cell nucleus.

We also note that Reviewers 1 and 2 have not criticized the wording.

Thus, we respectfully maintain that the term “protonucleus” is appropriate in the context of this work. The term denotes a previously established class of DNA-based synthetic compartments developed in our earlier studies, which were published in this journal[1].

We further want to distinguish the semantics issue from the platform-level claim. For us, the term “platform” indicates that we have a reusable, technical system at hand, that supports multiple assays such as the partition assay. The intended association with the term is that we have a scalable and repeatable system. We want to point out that we have since tested other proteins successfully within our platform and intend to pursue further projects with it. We also believe that the results of the manuscript demonstrate clearly the environmental impact on phase separation of proteins and thus extends beyond classical test tube assays.

To soften our claims in accordance with your comment, we have rephrased the quoted section from “an advanced and versatile test system between classical test tube/solution experiments and in cellulo studies” to the following:

"a bottom-up, programmable DNA-rich compartment system that complements classical test tube/solution experiments and *in cellulo* studies."

2) The new Figure 1 has some discrepancies from the previous version. The morphologies of the condensates are drastically different between the two versions. For instance, for the ratios 0.30 (new), the condensates appear larger in size after 5 hours compared to the ratio 0.37 (old) after 3-4 hours. The same morphological difference is true for the other regimes. More importantly, the "Exceeding Loading Capacity" is no longer observed in the new version and instead, "Inverted Region" is reported as the third regime for ratio 1.50. This level of inconsistency is concerning and the authors did not discuss it neither in the response nor in the manuscript. Moreover, in the revised manuscript, the whole notion of exceeding loading capacity is omitted. Was that observation wrong or was not reproducible? The authors need to clarify this. The same is true for the "Co-continuous Domain" that is now replaced by "Spinodal Domain" entirely without much similarity between the morphologies. In the rebuttal, the authors mentioned that they removed "Exceeding Loading Capacity" (page 22), though the authors need to clarify why they did not use HEPES for Figure 1 in the first place.

We acknowledge that there are differences between the old and new data sets. We want to outline in the following why the new data set is superior and why some aspects do not align between the two.

As the reviewer mentioned, the biggest difference is between the buffers used in both data sets. The exact compositions are:

Phosphate buffer (old data):
20 mM Na-Phosphate, 75 mM NaCl, 1 mM DTT, 2.5% glycerol, pH 7.5, residual $MgCl_2$ from PN stock.

HEPES buffer (new data):
10 mM HEPES, 200 mM KCl, 1 mM DTT, 5 mM $MgCl_2$, Glycerol 3%, pH 7.5

Since our experiments involve the co-phase separation of DNA (PN core) and FUS, we would expect a competition of the phosphate ions in the buffer with the backbone of the DNA, thus dampening the nucleic-acid induced phase separation. This explains why the old data includes a soluble domain, which is not apparent in the same experimental range for HEPES buffer.

Secondly, the two buffers differ in overall ionic strength, which shifts the phase diagram.

In our long-term experiments exceeding the time frame of one day, we have observed crystal formation in CLSM, which we attribute to the crashing out of magnesium phosphate. This is a common and expected outcome for phosphate buffers with magnesium ions present. While this did not occur in our initial short duration studies, such as Figure 1, it was problematic in our long duration experiments in Figures 2-6 and prompted us to change the buffer from phosphate to HEPES. Even though crystals were not microscopically observed in the old

dataset of Figure 1, the dissolved fraction of magnesium ions could have been lower because of the strong interactions with phosphate and seed formation. This difference in Mg^{2+} changes the ionic strength of the buffer and as a result also the expected phase behavior of the system. While we cannot quantify this difference, we believe it to be an additional argument for the apparent differences between both buffers.

In consequence, in the revision, we applied the buffer system used in the other figures.

The new data set provides ensemble views, time lapse views of the initial process and two time points for comparison of the early and late morphologies. What is evident from the time progression, for example, is that the "exceeding loading capacity" domain is morphologically very close to the end state in sample LR=1.50 of the new data. However, the distinction of the new data between early and late-stage morphology clearly allows the reader to assess the mechanistic difference.

Lastly, we want to point out to the reviewer that this new data is furthermore supported by the addition of the numerical simulations.

3) I still think that Figure 5 (formerly Figure 3) should include some quantification about the variability of the morphologies and condensate sizes with respect to the p(A20-m) ratios. I understand that the authors provided FRAP data to support the claims quantitatively, though the measure of size and morphology variations remain unanswered.

We thank the reviewer for this suggestion. We have now added a quantitative, single-PN image analysis in Supplementary Fig. 22 to assess how the fraction of p(A_{20}-m-XL) affects FUS phase separation and morphological heterogeneity.

Because the observed FUS morphologies range from discrete domains to interconnected networks and shell-like structures, an object-based condensate diameter or area cannot be defined consistently across all conditions. We therefore used two complementary quantitative descriptors that can be applied to all morphologies. First, the FUS-rich area fraction reports the fraction of the PN cross-sectional area classified as FUS-rich using a two-step image-segmentation procedure. Second, the coefficient of variation, calculated as the standard deviation divided by the mean FUS fluorescence intensity within each PN, reports the relative spatial heterogeneity of the FUS signal.

Both metrics quantitatively support the morphology assignments in the main text. In non-crosslinked PN, the FUS-rich area fraction decreases markedly while the coefficient of variation increases strongly over time, consistent with pronounced FUS phase separation and coarsening. At 25% crosslinking, the changes are more moderate, indicating reduced and partially arrested phase separation. At 50% crosslinking, only small changes are detected, consistent with weak phase separation, whereas the 75% and 100% crosslinked samples remain essentially unchanged over the course of the experiment. Because the analysis was

performed at the level of individual PN, the violin plots additionally reveal PN-to-PN variability. The distributions broaden most clearly under conditions showing pronounced phase separation, whereas they remain comparatively narrow in the highly crosslinked samples.

We believe that this additional quantitative analysis directly supports our previous image-based interpretation and demonstrates the progressive suppression of FUS phase separation with increasing PN-core crosslinking.

4) The revised Figure 6 addresses the earlier 3-day versus 4-day inconsistency and is much more informative than before. Nevertheless, terms such as “remains dynamic after 3 days” (the caption of the figure) are somewhat stronger than I would prefer given that the recovery remains “partial recovery” (as the authors added) rather than fully liquid-like behavior. Also, the new Figure 4 does not include the requested post-loading/pre-cleavage FRAP control, so the paper still cannot fully exclude the possibility that FUS loading changes core mobility before TEV addition. I recommend reducing the wording to “retains partial mobility” where appropriate and refine what the FRAP controls establish.

We thank the reviewer for this careful assessment. We agree that the phrase “remains dynamic after 3 days” may be too strong, because the FRAP data show partial recovery rather than fully liquid-like behavior. We have therefore revised the wording of the figure caption to “retains partial mobility”. This better reflects the data and avoids implying a fully liquid-like state.

However, we would like to clarify that the post-loading/pre-cleavage FRAP control requested by the reviewer is in fact included in the revised manuscript. In Figure 6a, the light-green traces explicitly labelled “MBP-FUS-GFP” correspond to the post-loading/pre-cleavage state, i.e. after MBP-FUS-GFP has been recruited into PN but before TEV addition and FUS phase separation. This is also stated in the figure caption. These data therefore provide exactly the requested reference for the mobility of loaded MBP-FUS-GFP prior to cleavage.

1. Xie, M., Chen, W., Vonk-de Roy, M. & Walther, A. Constructing synthetic nuclear architectures via transcriptional condensates in a DNA protonucleus. *Nat. Commun.* **16**, 8254 (2025).

Reviewer 4:

I co-reviewed this manuscript with one of the reviewers who provided the listed reports. This is part of the Nature Communications initiative to facilitate training in peer review and to provide appropriate recognition for Early Career Researchers who co-review manuscripts.

We thank the reviewer for their time and effort in evaluating our manuscript.
